# Perinatal high methyl donor alters gene expression in IGF system in male offspring without altering DNA methylation

**DOI:** 10.4155/fsoa-2016-0077

**Published:** 2016-12-13

**Authors:** Valérie Amarger, Fanny Giudicelli, Anthony Pagniez, Patricia Parnet

**Affiliations:** 1 UMR PHAN, INRA, Université de Nantes, 44000 Nantes, France

**Keywords:** DNA methylation, early growth, imprinted genes, methyl donors, nutritional programming, protein restriction

## Abstract

**Aim:**

To investigate the effect of a protein restriction and a supplementation with methyl donor nutrients during fetal and early postnatal life on the expression and epigenetic state of imprinted genes from the IGF system.

**Materials & methods:**

Pregnant female rats were fed a protein-restricted diet supplemented or not with methyl donor.

**Results:**

Gene expression of the *Igf2, H19, Igf1, Igf2r* and *Plagl1* genes in the liver of male offspring at birth and weaning was strongly influenced by maternal diet. Whereas the methylation profiles of the *Igf2, H19* and *Igf2r* genes were remarkably stable, DNA methylation of *Plagl1* promoter was slightly modified.

**Conclusion:**

DNA methylation of most, but not all, imprinted gene regulatory regions was resistant to methyl group nutritional supply.

## Lay abstract

Fetal environment influences fetal growth and may confer a risk to develop metabolic diseases, possibly through alterations in the epigenetic state of the genome. Imprinted genes constitute a special class of genes that are crucial for the control of fetal and postnatal growth and are closely associated with energy metabolism. In addition, these genes are finely regulated by epigenetic mechanisms that are themselves influenced by environmental factors. This study showed that methyl donor nutrients in maternal diet strongly influenced the expression level of imprinted genes in the liver of rat offspring, despite a mild effect on epigenetic regulation.

**Figure 1.**
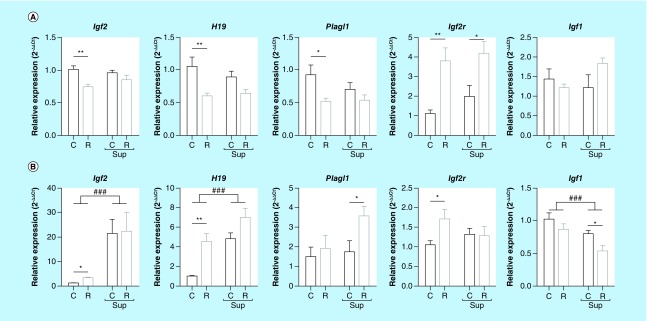
**mRNA expression of *Igf2, H19, Plagl1, Igf2r* and *Igf1* genes in offspring liver, (A) at D0, (B) at D21 (mean ± sem).** ^###^p < 0.001; ^####^p < 0.0001 (effect of methyl donor supplementation, two-way ANOVA). *p < 0.05; **p < 0.01. (Bonferroni *post hoc* comparisons) (n = 8/group).

**Figure 2.**
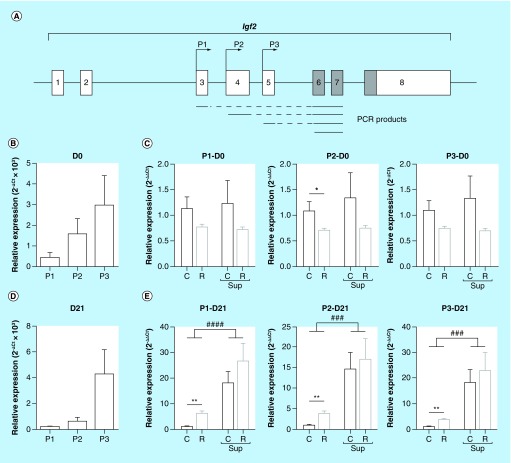
**mRNA expression of *Igf2-*specific transcripts expressed from promoters P1, P2 and P3 in offspring liver.** **(A)** genomic structure of the *Igf2* gene. Noncoding exons are represented by open boxes and coding exons by filled boxes. The position of the P1, P2 and P3 promoters are indicated by arrows above the exons. Polymerase chain reaction products used to quantify the promoter-specific or the total *Igf2* transcripts are presented below the gene structure. **(B & D)** Relative expression of the P1, P2 and P3 specific transcripts in the C group at D0 (**B**) and D21 **(D)**. **(C & E)** Relative expression of the P1, P2 and P3 specific transcripts in the four experimental groups at D0 **(C)** and D21 **(D)** at D21. Data are expressed as mean ± SEM (n = 8/group). ^###^p < 0.001, ^####^p < 0.0001 (effect of methyl donor supplementation, two-way ANOVA). *p < 0.05, **p < 0.01 (Bonferroni *post hoc* comparisons).

**Figure 3.**
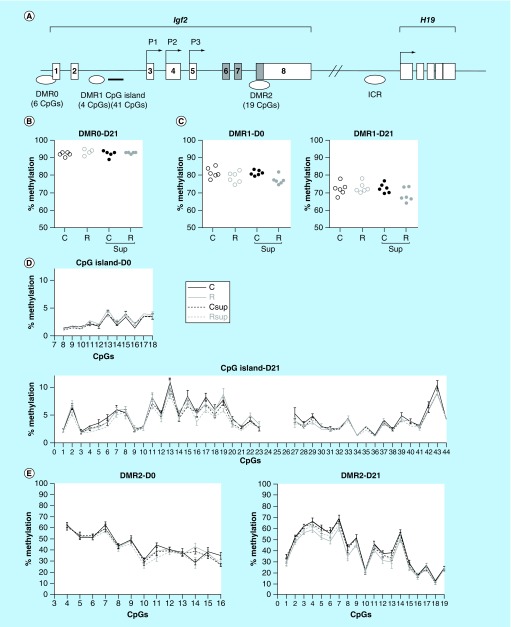
**DNA methylation in offspring liver at *Igf2* differentially methylated regions.** **(A)** Genomic structure of the *Igf2/H19* locus. Non-coding exons are represented by open boxes and coding exons by filled boxes. The position of the P1, P2 and P3 promoters are indicated by arrows. The positions of the different differentially methylated regions (DMRs), the intron 2 CpG islands and the imprinted control regions are indicated below the gene structure. **(B & C)** Methylation level of the DMR0 at D21 (n = 4–5/group), and DMR1 at D0 (n = 6/group) and D21 (n = 6/group). Each dot represents the average (mean) methylation level of six (DMR0) or four (DMR1) CpG sites for one specific DNA sample. **(D & E)** Methylation level of individual CpG sites from the *Igf2* intron 2 CpG island **(D)** and *Igf2* DMR2 **(E)** at D0 and D21 (mean ± SEM; n = 5–6/group).

**Figure 4.**
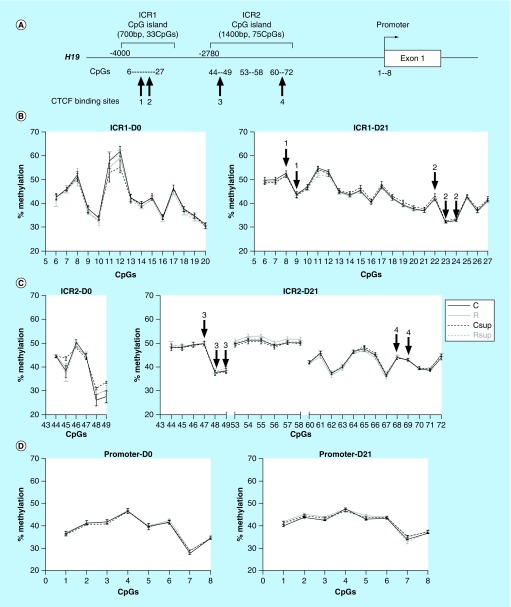
**DNA methylation in offspring liver at *H19* imprinting control region.** **(A)** Genomic structure of the *H19* upstream region. The position of the two CpG islands corresponding to the two parts of the imprinted control region (ICR) are indicated above the line. The CpG sites analyzed in this study are indicated below the line, as well as the position of the CTCF binding sites. **(B, C & D)** Methylation levels of individual CpG sites from the ICR1 **(B)**, ICR2 **(C)** and *H19* promoter **(D)** at D0 and D21. The CpG sites embedded in the CTCF binding sites 1 to 4 are indicated by arrows above the curves (mean ± SEM) (n = 6/group). CTCF: CCCTC-binding factor.

**Figure 5.**
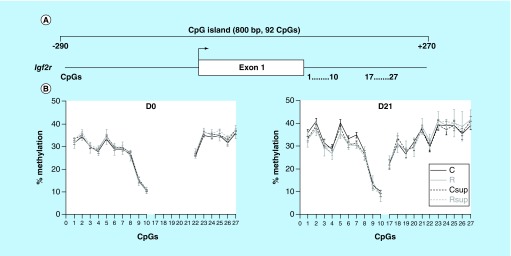
**DNA methylation level in offspring liver of the CpG island covering the promoter, exon 1 and part of intron 2 of the *Igf2r* gene.** **(A)** Position of the CpG island and of the CpG sites analyzed in this study. **(B)** Methylation level of individual CpG sites at D0 and D21 (mean ± SEM) (n = 6/group)

**Figure 6.**
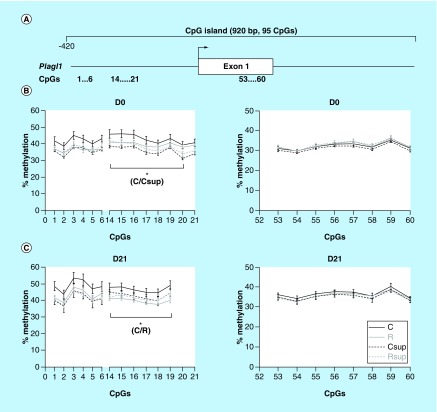
**DNA methylation level in offspring liver of the CpG island covering the promoter, exon 1 and part of intron 2 of the *Plagl1* gene.** **(A)** Position of the CpG island and of the CpG sites analyzed in this study. **(B & C)** Methylation level of individual CpG sites at D0 **(B)** and D21 **(C)**. *p < 0.05 (Dunn's *post hoc* test performed on the global mean of methylation levels for the considered CpG sites; mean ± SEM; n = 6/group).

**Figure 7.**
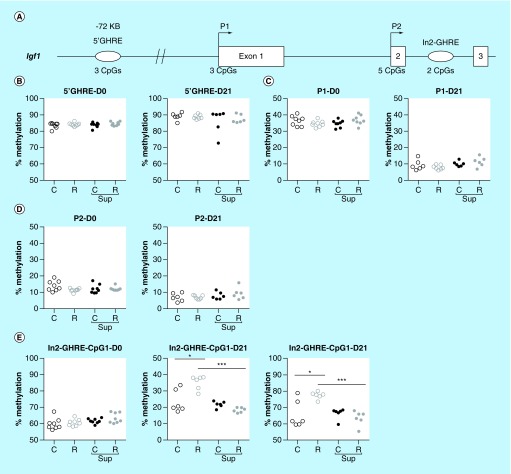
**DNA methylation level in offspring liver of the growth hormone responsive elements and promoters of the *Igf1* gene.** **(A)** Position of the analyzed regions relative to the *Igf1* gene, the exons are represented by open boxes, the promoters by arrows and the growth hormone responsive element (GHREs) by ovals. The number of CpG sites analyzed in this study is mentioned above the line. **(B–D)** Average methylation level of the 5′GHRE **(B)**, Promoter P1 **(C)**, Promoter P2 **(D)** at D0 and D21. Each dot represents the average (mean) methylation level of 3 (5′GHRE and P1) or 2 (P2) CpG sites for one specific DNA sample. **(D)** Methylation level of individual CpG sites in the intron2 (In2)-GHRE at D0 and D21. *p < 0.05, ***p < 0.001 (Dunn's *post hoc* test). (n = 6/group).

The concept of ‘nutritional programming’, initially based on observations on undernourished human populations [1–3] and cases of utero–placental deficiencies [4,5], suggests a link between fetal growth restriction and metabolic outcomes [6–9]. Animal models have been widely used to demonstrate that maternal nutrition can influence the epigenetic state of the fetal genome. This may constitute a major molecular mechanism linking early nutrition and altered gene expression and predispose to specific metabolic diseases later in life (for review [10–13]). Protein restriction is shown to be associated with altered methylation at a large number of genomic loci [14] or at specific promoters [15–21] and nutritional supplementation with one or several methyl donors (MDs) have been used to reverse the effects of protein restriction and were indeed shown to influence methylation profiles at specific loci (review in [22]). MD nutrients contribute to the one-carbon/MD pathway, critical for its role as provider of methyl groups for methylation reactions, including DNA methylation. Folic acid, methionine and choline are the main methyl group donors in most cell types, whereas betaine is used as an alternative MD in liver and kidney mostly [23,24]. The MD pathway relies on the activity of several enzymes that require additional cofactors, including vitamin B12 and zinc.

In humans, it is widely acknowledged that MD nutrients influence pregnancy outcomes and fetal health [25–27], and women who wish to be pregnant are currently advised to consume high amounts of MD, including folic acid. In addition, several countries have adopted a systematic fortification of basic food with folic acid and this policy has proven its efficiency for the reduction of birth defects such as spina bifida [28,29]. However, very few studies addressed the consequences of a high MD supply during pregnancy and of the possible interactions with the macronutrient content in maternal diet. And yet, several authors point out a link between systematic food fortification and an increased frequency of cancer, neurological disorders and possibly insulin resistance [30–32]. This raises questions about the potential negative consequences of an exposure to high MD during fetal and early postnatal life (for review [24]). For instance, high levels of folic acid during fetal life were shown to exacerbate the deleterious effect of a postweaning high-fat diet on metabolic outcomes in mouse male offspring [33]. A high multivitamin intake during pregnancy alters the control of food intake [34] and increases the risk to develop obesity in Wistar male rats [35] by affecting DNA methylation in hypothalamus [36]. MDs can therefore have wide and various effects on outcomes ranging from fetal growth and development to the programming of metabolic disorders.

Imprinted genes have proven to be highly interesting for their role in developmental programming [6,37]. These genes are characterized by a parent-of-origin-dependent mono allelic expression which mostly relies on epigenetic marks established in male and female gametes at specific differentially methylated regions (DMRs) and imprinting control regions (ICRs). Imprinted genes are strongly involved in the regulation of fetal and postnatal growth [38] and in the postnatal control of energy homeostasis, including adipose tissue differentiation [39], glucose/insulin related metabolism [40] and appetite regulation (reviewed in [41]).

Observations on human cohorts have shown that maternal periconceptual undernutrition [42] or altered fetal growth [43,44] may affect imprinted gene methylation and that a supplementation with folic acid, alone [45] or combined with zinc and vitamins [46,47] influences this effect. Regarding the methylation status of *IGF2* and *H19* genes in particular, the observed effects are very small in magnitude and inconsistent between studies that differed for a multitude of confounding factors, such as the genetic heterogeneity of human populations, the age of the studied individuals and the influence of multiple environmental factors (nutrition, stress). Studies on animal models make it possible to address the direct impact of specific nutrients without most of these confounding factors. However, studies conducted so far have shown variable and inconclusive results, depending mostly on the window of exposure and the nutritional changes [48–51]. The purpose of our study was to use a high MD supply in order to provide a nutritional challenge on the epigenetic machinery that requires methyl groups and address the lability/resistance of imprinted genes from the IGF system to this challenge.

We previously demonstrated in our model that MDs and proteins in maternal diet influence fetal and postnatal growth, insulin and leptin secretion [52] and neurodevelopment in the hippocampus [53]. Plasmatic homocysteine in male offspring at D21 was not affected by maternal diet. Postnatal growth and insulin secretion was impaired in offspring of protein restricted dams, whereas MD supplementation was associated with low plasma leptin at weaning. Male-only offspring from protein restricted-MD supplemented dams showed a reduced weight gain on control diet until the age of 23 weeks and remained 30% lighter than control males even after 4 weeks on western diet, despite a similar food intake, suggesting a difference in metabolic regulation or energy expenditure. We present here a unique and extensive study of the methylation profiles of DMRs, ICRs and promoter regions of several genes involving the IGF system in liver offspring.

## Materials & methods

### Selection of MD doses in experimental diets

Four experimental diets were designed to address the combined effect of protein and MD content in maternal diet. Protein restricted diets contained 8% protein (vs 20%) which is commonly used in many rodent models of nutritional programming ([12]. Protein restricted (R) or control (C) diets contained the recommended amounts of MDs and cofactors involved in methyl metabolism (methionine [7.2 g/kg in the C diet] choline chloride [1 g/kg], folic acid [2 mg/kg], zinc [30 mg/kg], vitamin B12 [25 µg/kg]) that are required during pregnancy and lactation [54], except for methionine that was reduced in the R diet (2.9 g/kg) because of global protein restriction. Two MD-supplemented diets [containing either 20% (Csup) or 8% (Rsup) protein] were designed to provide an increase in the total amount of both cofactors and MDs (×15 for choline, ×40 for vitamin B12, ×7 for folic acid, ×6 for zinc, ×1.6 [Csup vs C] or ×4 [Rsup vs R] for methionine and additional betaine [15 g/kg]) (see Supplementary Table 1 for detailed composition of the diets). These amounts of MD nutrients were chosen according to previous studies showing an absence of toxicity together with an impact of the methylation status of specific genes [55,56]. They were also below the doses used in studies addressing the impact of a single supplementation with high doses of methionine [57] or folic acid [33].

### Animals & diets

The protocol for animal procedures was approved by the local ethics committee for animal experimentation (Comité Régional d'Ethique en Experimentation Animale Pays de Loire) under the licence number CEEA.2010.02, and described previously [52,53]. Briefly, virgin female Sprague Dawley rats, 7 weeks old, weighing 200–220 g, were fed the experimental diets for 21 to 28 days before mating, and throughout gestation and lactation (six females per group). At birth (D0), a subset of pups was sacrificed and litters were culled to eight pups per dam (four males and four females). At D21 (after overnight fasting), another subset of pups were sacrificed. A maximum of three (Csup) or two (C, R, Rsup) pups from the same litter, and a minimum of three (Csup) or four to five litters per group were considered. At both time points (D0, D21) pups were sacrificed by decapitation and liver was quickly removed, snap frozen in liquid nitrogen and stored at -80°C. Only male pups were analyzed in the present study.

### Real-time quantitative reverse transcriptase-polymerase chain reaction

Total RNA was extracted from male offspring liver using Qiazol (Qiagen Sciences, MD, USA) according to the manufacturer's recommendations. Total RNA was quantified using the Nanovue spectrophotometer (GE Healthcare, France). RNA integrity was confirmed by agarose gel electrophoresis and, for a random set of samples, using the Agilent BioAnalyser 2100. cDNA synthesis and real-time polymerase chain reaction (PCR) were performed as previously described [53]. All samples were analyzed in duplicates and the cycle threshold (Ct) values were averaged. Gene expression was normalized using the geometric mean of the expression of two reference genes β-actin (*Actb*) and beta 2 microglobulin (*B2m*). The expression stability of these genes in liver was tested using the Genorm^®^ Software [58]. When comparing the relative mRNA expression between the four groups at a given time point, the expression was calculated with the 2^−ΔΔCt^ method using the control group (C) as reference condition. When comparing the expression between D0 and D21 (Supplementary Figure 1), or between the three promoters of the *Igf2* gene (Figure 2B & D) it was expressed as 2^−ΔCt^ × 10^3^. Expression changes were presented as the ratio of the means between two groups. Primer sequences are given in Supplementary Table 2.

### Pyrosequencing DNA methylation analysis

Genomic DNA was extracted from liver using the Nucleospin^®^ Tissue kit (Macherey Nagel, GmbH and Co, France). Two micrograms were submitted to bisulfite modification using the Methyl Detector bisulfite modification kit (Active Motif Europe, Rixensart, Belgium) according to the manufacturer's instructions. Bisulfite converted DNA was amplified using the Pyromark^®^ PCR kit (Qiagen Sciences) and pyrosequencing was performed using the Pyromark^®^ Q24 instrument (Qiagen Sciences) following the manufacturer's instructions. PCR and pyrosequencing primers were designed using the Pyromark^®^ Assay Design software (Qiagen Sciences). Primer sequences are given in Supplementary Table 3.

### Statistical analyses

Data were analyzed using GraphPad Prism^®^ 5 (GraphPad software Inc., CA, USA). Gene expression results (n = 8 per group) were analyzed using two-way ANOVA followed by Bonferroni *post hoc* comparisons when appropriate, in order to evaluate the impact of protein restriction and MD supplementation. Pyrosequencing data (n = 4 to 6 per group) were presented as absolute methylation levels of single CpG sites or the mean for several adjacent CpG sites (mentioned on the Figures 3– 7). Data were analyzed used the nonparametric Kruskal–Wallis test followed by Dunn's multiple comparison *post hoc* test. Significant effects (p-values < 0.05) were indicated in Figures 1, 2, 6 & 7).

## Results

### Maternal diet affected the expression of several imprinted genes at D0 and D21

The expression of the *Igf2, H19, Plagl1, Igf2r* and *Igf1 genes* was assessed by quantitative RT-PCR on mRNA extracted from male liver at D0 and D21. At D0, the expression levels of *Igf2, H19* and *Plagl1* were significantly reduced (0.74, 0.57 and 0.55-fold, respectively) in the R group compared with the C group, but this effect was no longer observed when the diets were supplemented with MD (Csup vs Rsup) (Figure 1A). To the contrary, *Igf2r* was overexpressed (3.45-fold) in the R group compared with the C group, and this effect was also observed when both diets were supplemented with MD. The *Igf1* expression did not vary either between the R and C groups, or between the Rsup and Csup groups.

The expression levels of *Igf2, H19* and *Plagl1* were strongly reduced at D21 compared with D0 (about 1000-fold for *Igf2* and *H19*) and, to the contrary, was higher for *Igf1 at D21* (Supplementary Figure 1). At D21, the two-way ANOVA revealed a strong effect of high-MD on the expression of *Igf2, H19* and *Igf1* (Figure 1B). *Igf2* and *H19* were overexpressed in the R group compared with the C group (3.17- to 4.52-fold, respectively) and strongly overexpressed in the Rsup and Csup groups (4.75- to 6.90-fold for *H19* and 19.82- to 20.76-fold for *Igf2*). *Plagl1* was slightly overexpressed in the Rsup group compared with Csup and *Igf2r* was overexpressed in the R group compared with C. The *Igf1* gene expression was influenced by the protein and MD content in maternal diet (two-way ANOVA). Whereas its expression was not different between C and R, it was significantly lower in the Rsup group compared with Csup, although the two-way ANOVA did not reveal any significant interaction. Since *Igf2* was overexpressed and *Igf1* was underexpressed in the high-MD groups, we tested whether their expression levels were correlated at D21. There was a significant inverse correlation (r = -0.50, p = 0.0025) between the expression levels of the two genes (data not shown).

### Maternal diet influenced the expression level of all *Igf2* promoters in liver

Since the *Igf2* gene is expressed from a set of alternative promoters [59] (Figure 2A), we tested whether the observed differences in the expression level at D21 were promoter specific or not. Three reference transcripts of the rat *Igf2* gene are present in the Nucleotide database [60] and the three promoter regions of these transcripts were similar to P1, P2 and P3 promoters in mouse [59]. *Igf2* was expressed predominantly from P3 at both D0 and D21, in all experimental groups. The proportion of transcripts expressed from P1 and P2 promoters at D0 was about 10 and 30%, respectively (Figure 2B), and this proportion fell to 4 and 12% at D21 (Figure 2D). This relative proportion, shown for the C group on Figure 2B & D was similar in the other groups. However, at D0, when comparing separately the expression of each transcript among the four groups, only the P2-specific transcript was significantly under expressed in the R group compared with the C group (Figure 2C). At D21, specific transcripts from all three promoters were overexpressed about threefold in the R group and between 15- and 30-fold in the Csup and Rsup groups compared with the C group (Figure 2E).

### *Igf2* DMRs displayed variable methylation levels that were not influenced by maternal nutrition

The *Igf2* DMR0 was identified by sequence similarity with the mouse genomic sequence. This region corresponds to the promoter region of the P0 placenta specific transcript and is known to have a parental specific methylation exclusively in placenta [61]. In liver at D21, six CpG sites from DMR0 were heavily methylated (92.6% ± 1.4) (Figure 3B) in all samples from the four experimental groups, which is consistent with the absence of expression of the P0 transcript (data not shown).

The intron 2 of the *Igf2* gene contains the DMR1 (Figure 3A), which is conserved with mouse DMR1 [62] but is less GC-rich in the rat. This region is immediately followed by a CpG island, known to contain a muscle-specific repressor element involved in the regulation of growth [63]. Four CpG sites from DMR1 were highly methylated in liver at D0 and D21, with a slight decrease between D0 (79.8% ± 3.0) and D21 (71.6% ± 3.5) (Figure 3C). To the contrary, the adjacent CpG island (Figure 4D) was hypomethylated at both D0 (16 CpG sites assessed, ranging from 0.5 to 5%) and D21 (41 CpG sites, ranging from 1 to 10%) (Figure 3D). There was no significant difference in the methylation level of these two regions between the four experimental groups.

*Igf2* DMR2 is situated at the 3′end of the *Igf2* gene and overlaps the start of the last exon (Figure 3A). This region is known to be methylated on the expressed paternal allele in mouse fetus [64]. The methylation level of this region in rat liver was rather heterogeneous from one CpG site to another, with methylation levels ranging from 30 to 65% at D0 and from 15 to 70% at D21 (Figure 3E) but there was no significant difference between groups.

### *H19* DMRs showed a typical imprinted gene methylation status which was not influenced by maternal diet

The GC-rich imprinted control region (ICR) situated from -4000 to -1000 relative to the *H19* transcription starting site (Figure 4A) is characterized by a paternal allele specific methylation. This region, strongly conserved among mammals, contains four binding sites for the vertebrate enhancer blocking factor CTCF and is essential for the control of imprint of *Igf2* and *H19* [65]. DNA methylation was measured for 15 (D0) to 22 (D21) CpG sites from the first part of the ICR (containing the first two CTCF-binding sites) and for 6 (D0) and 25 (D21) CpG sites from the second part of the ICR (containing the last two CTCF-binding sites). The methylation levels varied from 30 to 60% from one CpG to another but the methylation profiles throughout the two regions were strongly conserved from D0 to D21 and between the four experimental groups (Figure 4B & 4C).

The methylation level of eight CpG sites in the promoter region of *H19* was around 40% at both times with no difference between groups (Figure 4D).

### A CpG island spanning the 5′end of the *Igf2r* gene was not influenced by maternal diet

A large CpG island encompasses the promoter region, exon 1 and the beginning of the intron1 of the *Igf2r* gene (Figure 5A). Because of the high density of CpG sites in the promoter region, it was impossible to design pyrosequencing assays. Methylation level was measured for 21 CpG sites immediately downstream exon 1. The methylation levels varied from 10 to 40% at D0 and D21 and were similar between the four experimental groups (Figure 5B).

### The *Plagl1* promoter methylation was influenced by maternal diet

We analyzed the methylation level of 21 CpG sites from a large CpG island encompassing the promoter, exon1 and start of intron1 of the *Plagl1* gene (Figure 6A). This region is conserved with mouse and human and corresponds to a DMR common to the *PLAGL1* and HYMAI imprinted genes [66,67]. Thirteen CpG sites from the promoter region were about 40% methylated at D0 and between 40 and 50% at D21 (Figure 6B). At both times, the methylation level was higher in the control group. The averaged methylation level of the CpGs 14–20 was significantly lower in the Csup group compared with the C group at D0 (-7.1%) and in the R group compared with the C group at D21 (-7.05%). To the contrary, the methylation levels of CpG 53–60 were similar between the four experimental groups.

### A growth hormone response element in Igf1 intron 2 was hypermethylated in response to maternal protein restriction

Unlike *Igf2, H19, Igf2r* and *Plagl1* genes, the *Igf1* gene is not imprinted. However, its expression was shown to be associated with the methylation of several regulatory regions including growth hormone responsive elements (GHREs) and its two promoter regions P1 and P2 [47,68] (Figure 7A). Three CpG sites in the GHRE situated 72 kb upstream the *Igf1* gene were highly methylated at D0 (84.1% ± 1.4 on average) and at D21 (88.0% ± 3.9 on average), and there was very little variability between samples and between the three CpG sites (Figure 7B). Three CpG sites in the P1 promoter were on average 35% methylated (35.7 ± 2.6) at D0 in all groups but their methylation level fell to less than 10% (9.4 ± 2.5) at D21 (Figure 7C). Five CpG sites in the P2 promoter fell from 12% methylation (12.4 ± 2.3) at D0 to 7% (7.7 ± 2.5) on average at D21 (Figure 7D). For both promoters, there was no difference between groups. The CpG1 in the GHRE situated in intron 2 of the *Igf1* gene was highly methylated at D0 (61.5% ± 2.9, on average in all groups), and fell to 21.3% ± 4.5 at D21 in the C, Csup and Rsup groups, whereas it remained significantly more methylated (35.4% ± 4.2) in the R group (Figure 7E). The CpG2 from this GHRE was also hypermethylated in the R group compared with the three other groups.

## Discussion

The present study was designed to test the impact of a protein restricted diet and a supplementation with MD nutrients during gestation and lactation on the expression level and the methylation status of genes involving the IGF system.

We decided to focus our attention on genes that are both involved in the control of fetal growth and in the regulation of energy homeostasis, including insulin-mediated metabolism [40] and adipose tissue development [39]. IGFI and IGFII are two major growth factors regulating fetal and postnatal growth [69]. Their action is driven by a very complex set of regulations, including the control of gene expression and translation efficiency, the modulation of bioavailability and receptor competition [40,59,70]. For instance, the regulation of *Igf2* is closely associated with the expression of the neighboring noncoding *H19* gene [71]. Both genes are under the control of transcription factors among which the zinc-finger transcription factor encoded by the *Plagl1* gene [72] that acts through binding to a shared enhancer [67]. The IGFII-receptor, encoded by the *Igf2r* gene, acts as an antagonist of IGFII function by sequestering IGFII and targeting it for lysosomal degradation [73]. Therefore these two genes regulate the growth-promoting action of IGFII through two different ways, *Plagl1* at the gene expression level and *Igf2r* at the protein level.

The animal model used in this study was characterized previously [52]. Whereas the impact of maternal diet on birth weight and postnatal growth was rather similar between males and females, long-term impact on weight gain and especially when exposed to a hypercaloric diet, was observed in males only. This sex dimorphism in the susceptibility to respond to early nutrition is a known phenomenon evidenced both in human studies [74] and animal models [75]. Therefore, we decided to focus our study on male offspring.

### Protein restriction during gestation altered the expression of imprinted gene related to the synthesis and growth promoting action of IGFII & high MD attenuated this effect

The main effect observed at D0 in new born from protein restricted-low MD dams was a lower expression of the growth-promoting imprinted genes *Igf2* and *Plagl1* and an overexpression of the growth-restricting gene *Igf2r* in liver. The observed changes in *Igf2* and *Igf2r* mRNA expression levels may result in a reduced action of IGFII that could be an adaptive process to a reduced nutrient availability in the R group. *Igf2* was under expressed in rat fetal liver after a maternal low protein diet during the preimplantation period only [76] whereas an overexpression was observed by Gong *et al*. [49] using a similar low-protein diet from the second day of gestation until birth, and no change of expression in a mouse model of maternal 50% caloric restriction from day 12.5 of pregnancy [77]. These discrepancies in results certainly reflect the importance of the window of exposure and highlight the major impact of the periconceptual period. In our study, the dams were fed the experimental diets for 3 weeks before mating which is closer to the situation encountered in human when women are taking prenatal folic acid or when they are exposed to supplemented foods. In the present study, dams had adapted their metabolism to the diet before entering pregnancy, and that may explain the fact that, although the expression of the growth-promoting genes was reduced in the R group, the pups did not suffer from intrauterine growth restriction (IUGR) [52]. It is indeed now established that maternal nutrition may have long-lasting consequences on offspring metabolic outcomes without any impact on birth weight, both in humans [78] and animal models [12,79–80], and these consequences rely more on the time-window and nature of the nutritional insult than on growth restriction *per se* [80].

One interesting finding of our study was that high MD tended to attenuate the effect of protein restriction on the expression of *Igf2, H19* and *Plagl1* at D0, but not for the *Igf2r* gene. *Plagl1* is known to regulate the expression of these two genes in liver [72], therefore it is possible that it mediates the expression changes we observed for *Igf2* and *H19*. In addition, *Plagl1* DMR methylation was reduced in presence of high MD at D0, although the link between its DMR methylation and its expression level has not yet been established. In humans, the methylation level of the *Plagl1* DMR was shown to be associated with fetal and postnatal growth in healthy infants [81] and the expression of the gene was altered in IUGR placenta [67], strengthening our present results. Therefore, *Plagl1* may represent a major target through which maternal diet influenced the expression level of several genes.

### After D0, the switch from IGFII to IGFI for growth promotion was delayed by high-MD, possibly through epigenetic regulation of the In2GHRE

At D21, the *Igf2* and *H19* genes were strongly overexpressed in the high MD groups although their expression level was considerably reduced compared with D0. On the other hand, *Igf1* was under expressed at D21 in the high MD groups and the expression level of *Igf2* and *Igf1* were inversely correlated, suggesting that the normal transition of an *Igf2* to *Igf1* postnatal growth induction was somehow delayed in the presence of high MD. Considering that postnatal growth is mostly driven by *Igf1* [82], this finding is consistent with the observation that postnatal growth was strongly impaired in the Rsup group [52], which certainly suffered from the double impact of protein restriction and low IGFI.

The *Igf1* gene is not an imprinted gene but it obviously plays a major role in fetal and postnatal growth [82] and its expression is tightly dependent on epigenetic regulation that controls specifically its activation by growth hormone (GH) [47,83–84]. During postnatal life, circulating IGFI is mainly produced by the liver under the stimulation of GH but its ubiquitous production allows it to act through autocrine/paracrine mechanisms in every tissue [85]. Postnatal increased hepatic IGFI production is correlated to changes in histone marks and DNA methylation at the In2-GHRE, a potent enhancer of *Igf1* transcription, conferring to this genomic region an open chromatin state [68,86]. These changes, including a decrease in DNA methylation, were shown to be less pronounced in a model of IUGR induced by uterine artery ligature [68]. In line with these data, the In2-GHRE DNA methylation decrease between D0 and D21 was lower in our R group, suggesting that the nutritional insult was sufficient to induce that, independently of an effect on fetal growth. Surprisingly, the MD supplementation normalized the DNA methylation level at D21, but this was not sufficient to restore expression levels similar to controls, possibly because the other epigenetic marks were not normalized or because of a state of GH resistance [87]. A better understanding of the current mechanism would require more investigations, in addition to the quantification of IGFI and GH plasma levels.

Our present study was not designed to test if the lower expression of *Igf1* was beneficial or not in the long term. However, the slowdown growth observed in Rsup rats was still noticeable at adulthood and conferred them a lower weight gain under western diet despite a similar food intake [52]. The role of the epigenetic regulation of *Igf1* in the protective effect against metabolic morbidities of a reduced postnatal growth after a nutritional restriction during fetal life was already suggested [88]. Our data reinforce this primary hypothesis and go further by suggesting that high-MD may enhance this effect, even though it remains to be determined whether this effect was due to the pre- or postnatal MD supplementation.

### The methylation profiles of most DMRs were not impacted by maternal nutrition

We showed here that, despite significant changes in expression levels of several imprinted genes, only the *Plagl1* DMR methylation level was influenced by maternal diet. Although studies on human cohorts have emphasized the effect of maternal nutrition on the methylation level of imprinted genes’ DMRs [42,46,81,89–92], the demonstration of such effect on animal models still remains to be established. In agreement with previous results on a mice model of maternal protein restriction [50,76], we found that, for the *Igf2, H19* and *Igf2r* genes, changes in the expression level are not related to alteration in the methylation pattern of the DMRs or promoters. This study was the first to address such a large number of CpG sites from the various DMRs of the *Igf2* gene and to reveal both a high diversity of methylation status from one CpG to another inside a DMR and a great homogeneity between individuals. These observations indicate a narrow and strong control of the methylation patterns that is resistant against potential environmental disturbances. The reason might be that an unaltered methylation pattern of imprinted genes is mandatory to ensure a normal feto–placental development [93] or that these genomic regions do not go through the demethylation wave that occurs just after fertilization [94].

A number of issues may be raised regarding our study, as it is the case for most animal models dealing with nutritional interventions. The protein restriction model results in the shortage of all amino acids which may influence a wide range of metabolic parameters. For instance, methionine is both involved in protein synthesis and in one-carbon metabolism [95]. Regarding MDs, we cannot conclude that the observed effects are not due to a specific nutrient rather that the combination of them. For instance, zinc is known to be involved in a wide range of biological functions, although its role during pregnancy remains elusive [96]. The purpose of our model was to increase the availability of methyl groups, whose main nutritional providers are folic acid, methionine and choline [97,98].

In addition, the choice to use a rat model and the pyrosequencing technology for DNA methylation quantification did not allow assessing allele-specific methylation, which would be potentially informative. However, the rat model of nutritional programming is widely used because it is more appropriate than mice for a number of physiological characteristics and pyrosequencing is recognized as one of the most accurate method for DNA methylation analyses.

## Future perspective

We showed here that protein and MD content in maternal diet influenced the expression level of several imprinted genes in offspring liver at birth and weaning in the absence of modifications in the methylation status of imprinted DMRs. The *Plagl1* gene was the only imprinted gene to show altered methylation level in response to maternal diet. Since this gene encodes a major transcription factor, further investigations are certainly required in order to address whether the *Plagl1* gene constitute a specific target through which nutrients impact the expression level of a large number of genes. Additionally, we showed that MD supplementation was capable to restore the normal postnatal methylation decrease at the *Igf1* In2-GHRE which was altered by protein restriction. Finally, we propose that the reduced postnatal *Igf1* expression, induced by the combined effect of protein restriction and MD supplementation, may be the mechanism that minimizes the weight gain of our adult animals after slowing down their postnatal growth.

**Executive summary**

Folic acid supplementation is highly recommended at the beginning of pregnancy and, associated with systematic food fortification and/or the use of multivitamins supplements may be responsible for high amounts of methyl donor (MD) micronutrients. The impact on the fetus and the interaction with macronutrient content in maternal diet are still poorly known.
The IGF I and II are involved in fetal and postnatal growth and postnatal control of energy homeostasis. They may constitute major targets in early nutritional programming, possibly through an altered epigenetic regulation.
The expression level of several imprinted genes from the IGF system was altered in the liver of offspring in response to the amount of protein and MDs in maternal diet during the gestation and lactation periods. A high MD content tended to reduce the effect of protein restriction during gestation.
The methylation level of the *Igf2, H19* and *Igf2r* gene regulatory regions was remarkably stable in the liver of offspring. The *Plagl1* gene promoter region was hypomethylated in response to MD supplementation or protein restriction at D0 and D21, respectively.
