# Supplementary material for: Perinatal high methyl donor alters gene expression in IGF system in male offspring without altering DNA methylation
Source: Future Sci OA. 2016 Dec 13;3(1):FSO164. doi: 10.4155/fsoa-2016-0077 (PMC5351714; doi:10.4155/fsoa-2016-0077)
Supplement: Supplementary file 1 [file fsoa-03-164-s1.docx]

**Supplementary Table 1**: Detailed composition of the experimental diets

|  | Control (C) | Control-MD supplemented  (Csup) | Protein Restricted (R) | Protein Restricted-MD supplemented (Rsup) |
| --- | --- | --- | --- | --- |
| Dextrose (%) | 10 | 10 | 10 | 10 |
| Sucrose (%) | 10 | 10 | 10 | 10 |
| Soybean oil (%) | 4.3 | 4.3 | 4.3 | 4.3 |
| Cellulose (%) | 5 | 5 | 5 | 5 |
| Corn Starch (%) | 43.6 | 37.8 | 56.6 | 50.3 |
| Casein (%) | 22 | 22 | 9 | 9 |
| Methionine (g/kg) | 7.2 | 12 | 2.9 | 12 |
| Choline chloride (g/kg) | 1 | 15 | 1 | 15 |
| Betaine (g/kg) | 0 | 15 | 0 | 15 |
| Vitamin B12 (µg/kg) | 25 | 1000 | 25 | 1000 |
| Folic acid (mg/kg) | 2 | 15 | 2 | 15 |
| Zinc (mg/kg) | 30 | 180 | 30 | 180 |
| Energy (kcal/kg) | 3260.8 | 3064.6 | 3261.6 | 3051.4 |
