# Supplementary material for: Perinatal high methyl donor alters gene expression in IGF system in male offspring without altering DNA methylation
Source: Future Sci OA. 2016 Dec 13;3(1):FSO164. doi: 10.4155/fsoa-2016-0077 (PMC5351714; doi:10.4155/fsoa-2016-0077)
Supplement: Supplementary file 2 [file fsoa-03-164-s2.docx]

**Supplementary Table 2**: Sequences of primers used for RT-PCR analyses.

| **Gene** | **Orientation** | **Sequence 5’-3’** |
| --- | --- | --- |
| Igf2 (all isoforms) | Forward | AAGTCGATGTTGGTGCTTCTC |
|  | Reverse | GAAGGCCTGCTGAAGTAGAA |
| Igf2 P1 | Forward | TCCCACTTCTGCAGCTCTC |
|  | Reverse | GAAGGCCTGCTGAAGTAGAA |
| Igf2 P2 | Forward | GTTTGCATACCCGCAGCAG |
|  | Reverse | GAAGGCCTGCTGAAGTAGAA |
| Igf2 P3 | Forward | CCTGTGAGAACCTTCCAGC |
|  | Reverse | GAAGGCCTGCTGAAGTAGAA |
| H19 | Forward | CCCTCAAGATGAAAGAAATGGTG |
|  | Reverse | AGGTAGTGTAGTGGTTCTGAG |
| Plagl1 | Forward | ATCCACAATTATTCCCACACCA |
|  | Reverse | CGTAGCCATGTGTCTCATCAG |
| Igf2r | Forward | TGTATCCGTGAACCTGTGTC |
|  | Reverse | AGTTGTCCTCTTCCTGATATTCTG |
| Igf1 | Forward | AAGCCTACAAAGTCAGCTCG |
|  | Reverse | GGTCTTGTTTCCTGCACTTC |
| Actb | Forward | CTATCGGCAATGAGCGGTTCC |
|  | Reverse | GCACTGTGTTGGCATAGAGGTC |
| B2m | Forward | CTGGTCTTTCTACATCCTGGCT |
|  | Reverse | TACATGTCTCGGTCCCAGGT |
