# Supplementary material for: Perinatal high methyl donor alters gene expression in IGF system in male offspring without altering DNA methylation
Source: Future Sci OA. 2016 Dec 13;3(1):FSO164. doi: 10.4155/fsoa-2016-0077 (PMC5351714; doi:10.4155/fsoa-2016-0077)
Supplement: Supplementary file 3 [file fsoa-03-164-s3.docx]

**Supplementary Table 3**: Sequences of primers used for Pyrosequencing analyses.

| **Gene/ genomic region** | **Function *** | **Sequence 5’-3’** |
| --- | --- | --- |
| *Igf2* / DMR0 | Forward | GAGTAGAAGTTATTTTTAGGGG |
|  | Reverse (biot) | TATTAACAACCTCCTCCAAAACAAAAAATAC |
|  | Sequence | GTTTTTTTAGATTTAGTTT |
| *Igf2* / DMR1  (CpG 1-2) | Forward | AGGGAAAGGAGAGGGTTTTAGAT |
|  | Reverse (biot) | CTATATAACTACCCACAAAACCTTCAC |
|  | Sequence | AGAGGGTTTTAGATAAAGAAT |
| *Igf2* / DMR1  (CpG 3-4) | Forward (biot) | TGTGGGTAGTTATATAGAGGAAGAAG |
|  | Reverse | CCTAATCTCTACTTTCTACCCTACAA |
|  | Sequence | CTACAAAACCACACCC |
| *Igf2* / CpG island (CpG 1-23) | Forward | GGGTTTTTAAATTGAGTTAGGGA |
|  | Reverse (biot) | AAATTTCCCCTTCTAAACAACC |
|  | Sequence (CpG 1-13) | GGGTTTTTAAATTGAGTTAGGGA |
|  | Sequence (CpG 14-23) | GTAGGGGTAGTGGGGAAT |
| *Igf2* / CpG island (CpG 27-44) | Forward | GGTTGTTTAGAAGGGGAAATTTT |
|  | Reverse (biot) | CTAAATTTAACAAACTAAACTCCC |
|  | Sequence | AAATATAGTTGGTTTAGG |
| *Igf2* / DMR2 | Forward | TTTGAAATTTGTTGATTAGTATTTTT |
|  | Reverse | CCCTACTCAAAAAAAAATCACAA |
|  | Sequence (CpG 1-3) | TTTGAAATTTGTTGATTAGTATTTTT |
|  | Sequence (CpG 4-11) | GATATTTGGAGATAGTT |
|  | Sequence (CpG 12-16) | TATGTTTGTTAAAGAGTT |
|  | Sequence (CpG 17-19) | TGTTATTATTTAAAGATTT |
| *H19* / ICR1  (CpG 6-12) | Forward | GAGTTATTAAGAGGTATGAGGATTATGT |
|  | Reverse (biot) | ACCCACAATATTACCTTATATAAATTCC |
|  | Sequence | GAATTTGTTGTGTGGTT |
| *H19* / ICR1  (CpG 13-27) | Forward | AGGGTAGGATATATGTATTTTTAGGT |
|  | Reverse (biot) | AACTCATAAAACCCATAATTATAAAATCA |
|  | Sequence | GGATATATGTATTTTTAGGTTGG |
| *H19* / ICR2  (CpG 44-49) | Forward | GTTTTTGGATTTTTAAATTAGTTAG |
|  | Reverse (biot) | TCCCCAAAAATTAACTACTTCTAAACT |
|  | Sequence | GTTTTTGGATTTTTAAATTAGTTAG |
| *H19* / ICR2  (CpG 53-58) | Forward | TTTTGGAGTGGTTGTATATTGA |
|  | Reverse (biot) | TACCACCCCATAACCCTTATAA |
|  | Sequence | GATTTATTTTTTATATAGTT |
| *H19* / ICR2  (CpG 60-72) | Forward | TATAAGGGTTATGGGGTGGTAGGATAT |
|  | Reverse (biot) | CCAAATACCTAACCCCTTTATT |
|  | Sequence | GTAGGATATATATTTTT |
| *H19* / promoter | Forward | TTGTTTAAGGGATTTTAAAGTGGGAGTTG |
|  | Reverse (biot) | CCCCTACTCTATCAACCAATCAAT |
|  | Sequence | AGGTTGTTTTTGGAGAA |
| *Igf2r* / intron 1 | Forward (biot) | TTTTGTGTAGGTGGGTGTTT |
|  | Reverse (biot) | CTCCTAAACCCAACTACTAAAACTTC |
|  | Sequence | CCCCACTTATAACAACCACAAAT (with Forward biot) |
|  | Sequence | GGTTATTTGTGGTTGTTATAAGT (with Reverse biot) |
| *Plagl1* / Promoter | Forward | GAGTTTTTTTGGTTTTTGGTTATAAGT |
|  | Reverse(biot) | TACTCCCCCCCCCCATTC |
|  | Sequence (CpG 1-6) | ATTATTTTTTTTTTTTAGTTTTTAT |
|  | Sequence (CpG 14-21) | GTATTTAGGAGATTTTGGTTGTG |
| *Plagl1* / exon 1 | Forward | ATTTTTTTGGTTGGTTTGTTG |
|  | Reverse (biot) | CAACACCAAAACTATCACTTACCT |
|  | Sequence | TTTTGAGAGTAGTTATGGTTGTTTA |
| *Igf1* / 5’GHRE | Forward | AGAGAGGTGGTAAGAGAATTTT |
|  | Reverse (biot) | CAAATTTTAACCCAAAACTTAAAAAAATAT |
|  | Sequence | ATAATTTGTAAAATAATAGGTTGTT |
| *Igf1* / P1 | Forward | TGGTTAGGTGTATTAGTAGATAAGTG |
|  | Reverse (biot) | ACAAATAACATCATACCTTTACTTTT |
|  | Sequence | GTGTATTAGTAGATAAGTGTATTT |
| *Igf1* / P2 | Forward | GGGATTGTGGAATGTTATTTTAGTAGGTA |
|  | Reverse (biot) | AACAACTCCCTTCAACCATCTCCTA |
|  | Sequence | GAATGTTATTTTAGTAGGTATTTAT |
| *Igf1* / In2-GHRE | Forward | TGGAATGTTATTATAGTTTTTGTTTGTTTT |
|  | Reverse (biot) | CTACCAAAAAACCCTTAATTAACACA |
|  | Sequence | GTTTTTTAGAATGAAGAGAGAT |

*Forward and Reverse primers for PCR, Sequence primers for Pyrosequencing reaction

(biot)-5’ biotin for purification before Pyrosequencing reaction
