# Supplementary figures and images for: Perinatal high methyl donor alters gene expression in IGF system in male offspring without altering DNA methylation
Source: Future Sci OA. 2016 Dec 13;3(1):FSO164. doi: 10.4155/fsoa-2016-0077 (PMC5351714; doi:10.4155/fsoa-2016-0077)

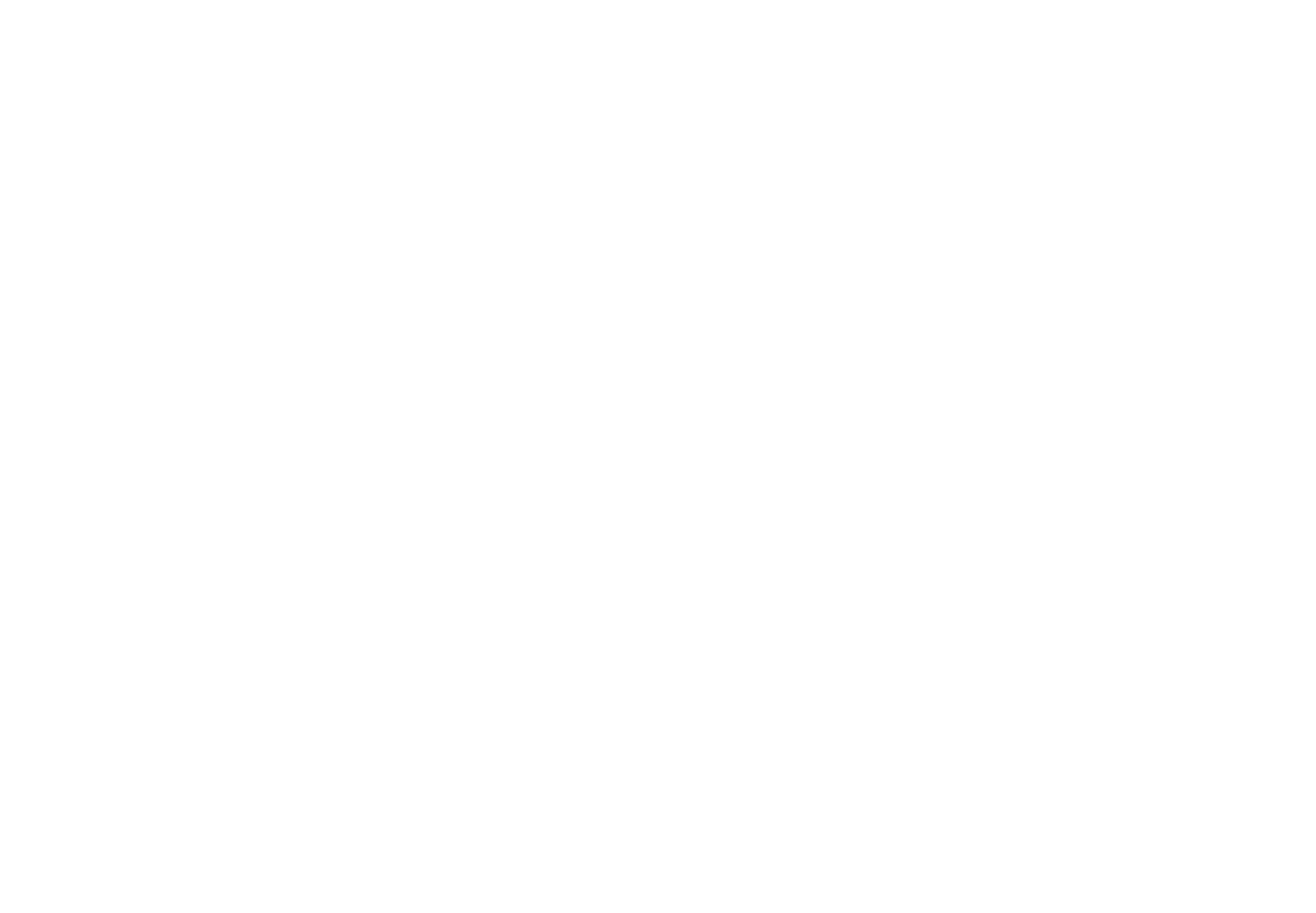

Supplement: Supplementary file 4 [file fsoa-03-164-s4.tif]
